# Do the Redundant and Locally Dependent Items of the LS/CMI Contribute in Any Meaningful Way to Its Reliability and Its Potential to Predict Criminal Recidivism?

**DOI:** 10.1177/0306624X231212815

**Published:** 2023-12-01

**Authors:** Guy Giguère, Christian Bourassa

**Affiliations:** 1 Université Laval, Québec, QC, Canada; 2 Université de Montréal, Pavillon Marie-Victorin, QC, Canada

**Keywords:** alpha index, Rasch, reliability, dependence locale, LS/CMI, validity

## Abstract

This article studies the effects of local dependence within the items of the first section of the LS/CMI on its reliability. Analysis were done to identify the dependent items namely through their correlations before and after Rasch modeling. Seven items were thus discarded, deemed dependent and redundant, and Cronbach’s alpha was calculated with all 43 items and then with the 36 items deemed independent. Test information and predictive validity were also compared. Removing the seven redundant items did not seem to have major effects on the reliability of the LS/CMI or the psychometric information it provided, and no tangible effects were observed on its predictive validity. The reliability of an instrument should be assessed with items that contribute each in its own way. However, it is hazardous to report the reliability of an instrument known to be multidimensional with means meant to be used with unidimensional instruments.

## Introduction

The assessment of risk factors is a central concern for criminal justice and correctional services (Gottfredson & Moriarity, 2006; Harcourt, 2007; Makarios & Latessa, 2013). Assessment approaches and tools have gone through different phases in their evolution. Bonta and Andrews (2016) propose four generations of risk assessment approaches. The first generation, which dominated much of the twentieth century, relied on experience and clinical judgment. This approach was informal and subjective (Bonta & Andrews, 2007) and the predictive potential of tools developed under this approach only exhibited a slight improvement over chance, as reported in Douglas and Skeem’s (2005) meta-analysis. Second-generation tools considered historical elements such as the precocity, nature, and frequency of criminal history. The meta-analysis by Ægisdóttir et al. (2006) found that second-generation actuarial tools were more accurate than a professional’s clinical judgment in terms of predictive validity. However, tools of this generation faced two shortcomings: they were considered “atheoretical,” and the wording of their items labeled them as static, and therefore unchangeable (Bonta & Andrews, 2007), while a new paradigm was slowly emerging; one based on the premise that offenders could change their offending behavior if their criminogenic needs were assessed and addressed. The Static-99 (Hanson & Thornton, 2000) is an actuarial tool used to assess the risk of recidivism among sex offenders, and it is composed of static items only. Thus, third-generation risk assessment instruments (e.g., Andrews & Bonta, 1995; Hanson et al., 2007) started incorporating dynamic factors that considered changes in an offender’s situation, while providing practitioners with information about the needs that should be targeted in their interventions, following the offender assessment and rehabilitation model of Andrews and Bonta (2006) based on the principles of risk, need, and responsivity (R-N-R). Within this model, the risk principle claims that criminal behaviors can be predicted, the need principle focuses on the importance of studying the criminogenic factors behind an individual’s behaviors, and the responsiveness principle refers to the offender’s capacity to benefit from the treatment. The LSI-R represents this generation of actuarial tools. Finally, a fourth generation of actuarial tools emerged in the 2000s. This latest generation of tools goes beyond its predecessors by integrating systematic intervention and monitoring with the assessment of a broader range of offender risk factors that were previously not measured, as well as combining other personal factors central to treatment (Bonta & Andrews, 2007). The primary aim of these tools is to gather such comprehensive information to facilitate case management throughout the entire process from intake to case closure. One prominent example of a fourth-generation tool is the Level Service/Case Management Inventory (LS/CMI). In this article, we propose a quantitative analysis of the LS/CMI from a novel perspective.

Despite the numerous validity studies that have allowed this generational classification of actuarial tools to be established, there is not much psychometric literature regarding the internal consistency of actuarial tools. According to Helmus and Babchishin (2017), it is natural since such criterion-referenced scales are assembled with “the smallest number of items measuring the most distinct constructs as possible.” As such, they are often atheoretical, designed to address one single outcome, not to fit one’s profile into an established framework. The Static-99R which includes ten items (Hanson & Thornton, 2000), the B-SAFER which includes a list of 10 risk factors and an interview guide (Storey et al., 2013) and the VRAG which includes twelve items (Quinsey et al., 2006) are good examples of such scales. With only a few items that are not expected to correlate, actuarial tools cannot be expected to show outstanding internal consistency. When they do, they most likely include redundant items (Helmus & Babchishin, 2017).

The LS/CMI is not such an atheoretical scale. It is anchored on the general personality approach and social learning theory of Andrews and Bonta (2006). Furthermore, it comprises several items—43 in the first section—many addressing the same constructs and its reliability through internal consistency is regularly reported. Its authors—Andrews et al. (2010)—argue that the internal consistency (Cronbach’s alpha) for all items of the LS/CMI varies between .88 (good) and .92 (excellent) depending on the studies and that the observed mean of the alpha indices within ten different studies is .89 with a .95 confidence of [.87, .91]. However, with many items pertaining to the same constructs, the internal consistency of the LS/CMI could be inflated by the presence of redundant items. Such items, carrying the same information and, de facto, exhibiting very strong correlations (Giguère et al., 2015), could weigh more in the calculation of Cronbach’s alpha, as Laurencelle (1998) suggested. Caution is advised when interpreting Cronbach’s coefficient; while it can be the product of desirable correlations among items, it can also be increased artificially by redundant items whose correlations defy reason. The purpose of this study is to investigate how the redundant items of the LS/CMI affect its internal consistency. To this end, Cronbach’s coefficient will be calculated with all its items and, ultimately, with fewer items once redundancy has been dealt with.

### Cronbach’s Alpha

Cronbach’s alpha is a measure of internal consistency and reliability referring to the degree to which items measure the same concept (Cronbach, 1951). This coefficient varies between 0 and 1 and the nearer the index is to 1, the more likely the items seem to be measuring the same construct.

Different thresholds for alpha are said to be acceptable; in truth, the acceptable thresholds as well as the very meaning of the alpha coefficient will vary according to the type of construct being measured and the number of items the instrument comprises (Walsh & Betz, 2001). Yet, acceptable values are usually found between .7 and .9. It should be noted that an alpha coefficient that is very high can be as undesirable as a very low alpha (Churchill & Peter, 1984), one reason being that it can hide interdependence between items (Sijtsma, 2008). Within the framework of item response theory (IRT), this interdependence is known as local dependence.

Also, an instrument comprising several items will likely have higher internal consistency than similar instruments comprising fewer items, simply because of how alpha is calculated. Cortina (1993) showed that a 40-items test assembled with uncorrelated items could have a sufficiently high alpha coefficient ( a=.7 ) based on the number of items alone. Thus, the reliability of a scale is somewhat proportional to its length (Streiner, 2003) and claiming that a test is reliable because of alpha’s value could be misleading because the length of an instrument is not psychometric evidence.

### Local Dependence

According to Yen (1993, p. 188), “[when] a pair of items is locally independent, the conditional probability, given the student’s ability level, θ, of obtaining any pair of scores on these items is the product of the probabilities for the separate items.” Interdependent items, whose endorsement prescribes another’s, have higher conditional probabilities. Since one interpretation of coefficient alpha represents the correlation of all possible split-halves, including those where two interdependent items are in different halves, their presence does, ultimately, inflate coefficient alpha’s value (Zenisky et al., 2001).

Most IRT models assume local dependence to have been accounted for, thereby respecting the local independence postulate. Local independence assumes that one’s performance or endorsement of each item of a measuring instrument is not influenced by his performance or endorsement of other items in the same instrument (Bertrand & Blais, 2004). This means that the endorsement of an item should only depend on the trait being measured. Thus, the notion of local independence has certain relationships with unidimensionality, another postulate within IRT. It can also be shown that when the assumption of unidimensionality is met, so is, usually, the assumption of local independence (Bertrand & Blais, 2004, p. 201).

High correlation does not necessarily entail local dependence, but local dependence will naturally yield high correlations. Linacre (2022a) suggests not to be concerned about dependence until correlation coefficients exceed .7. At this point, 2 items start having more than half their variance in common ( $r^{2}=.7^{2}=.49$ ).

Locally dependent items are redundant; they duplicate information that is already known, to no avail. Although the LS/CMI shows high internal consistency and desirable predictive validity, it does contain many items that must be endorsed for others to be. However, Baird (2009, p. 8) affirmed that “for risk assessment, it is best when all risk items are totally independent of each other, but each has a relatively strong relationship to the outcome measure utilized. These principles are in direct conflict with maximizing Cronbach’s alpha.” Furthermore, he adds that “measures of internal consistency, while important in psychology, are not relevant to risk assessment in corrections” (Baird, 2009, p. 8). Yet, despite its vicissitudes, Cronbach’s alpha remains the most widely used measure of scale reliability (Bourque et al., 2020; Sijtsma, 2008; Streiner, 2003).

### Aims of the Study

In this paper, we wish to show how the alpha coefficient can be influenced by redundant or locally dependent items, namely items measuring the same subconstructs. Section 1 of the LS/CMI—“General Factors of Risk Factors/General Needs”—is filled with items that are intertwined, whose endorsement follows or prescribes another’s (Giguère & Lussier, 2016; Giguère et al., 2015). Within modern psychometric models (e.g., Rasch, Item Response Theory), this is referred to as local dependence and such conditions must be avoided or tended to unless solid arguments are made to justify condoning them.

Few empirical studies have been devoted to examining the effects of this dependence on the reliability and the predictive validity of an actuarial tool. Giguère et al. (2015) studied the psychometric properties of the 43 items that constitute section 1 of the LS/CMI. They identified items that are problematic, either because they do not discriminate enough or because they seem redundant. However, they did not elaborate on the effects of this redundancy as we aim to do in the current study. In sum, we wish to estimate the internal consistency of the LS/CMI once rid of its locally dependent items. Hence, this evidence for reliability should be more representative of the “true” internal consistency of the LS/CMI. Also, we wish to observe how local dependence affects the predictive validity of the LS/CMI by comparing the coefficients calculated with all 43 items and with only the locally independent items of section 1 of the LS/CMI.

## Methodology

### LS/CMI Actuarial Tool and the French Version of the LS/CMI

The LS/CMI is a fourth-generation actuarial tool used to assess and manage the risk posed by offenders (Andrews et al., 2004). It includes 11 sections, but only the 43 items comprising section 1 are used for calculating the risk score. They are all scored dichotomously (1 for endorsing, 0 for not endorsing), yielding a total risk score that ranges from 0 to 43. Those 43 items are distributed in 8 theoretical dimensions: Criminal History (8 items); Education/Employment (6 items); Family/Marital (4 items); Leisure/Recreation (2 items); Alcohol/Drug Problem (8 items); Procriminal Attitude/Orientation (4 items); and Antisocial Pattern (4 items) (Andrews et al., 2004).

The LS/CMI was implemented in Quebec when the new law (Act regarding Quebec’s correctional system) came into force in 2007. It was translated into French for use in Quebec correctional services. According to sections 12 to 15 of this law, Quebec correctional services are required to assess each convicted person at the start of the sentence. Those evaluations are conducted by a probation officer or a prison counselor that interviews the individual and consults his criminal record. The information gathered is used to assess the individual’s criminogenic factors, his risk of reoffending, his potential for social reintegration and to constitute his correctional intervention plan. Since Quebec’s correctional services only manage short sentences, the timeframe is too small to allow other assessments.

### Sample and Data Collection

The sample used for this study contains the LS/CMI entries of 15,961 convicted and incarcerated men from Quebec, registered, and evaluated between March 2008 and March 2015, along with criminal recidivism data with a two-year follow-up. The individuals within this sample all received a sentence of less than 2 years, meaning that they were under the responsibility of Quebec’s Department of Public Safety.

### Analytical Strategy

To investigate the effects of local dependence within the items of the LS/CMI on its internal consistency, different analyzes were conducted. First, the Pearson correlations between the items of the LS/CMI were produced. Correlations considered “too” high (r≥0.7) were highlighted. Clusters of items deemed suspicious were not further analyzed or manipulated at this stage, additional evidence had to be collected to adequately identify dependent items. Then, the LS/CMI data was fitted into a Rasch (1960) model. Rasch modeling yielded estimates for items (difficulty) and individuals (ability) on the first and most explanatory dimension among those a PCA would reveal. However, for this study, neither parameter was of interest.^1^ The useful information was found at the residual level once the effects of the Rasch dimension were dismissed. Using Winsteps (Linacre, 2022b), table 23.99 (Largest residual correlations for items) was produced, showing which residual values seemed highly correlated, possibly indicating dependence and redundancy.

Next, clusters of items that seemed highly correlated before considering the effects of the Rasch dimension, and whose residual values are also highly correlated passed the Rasch dimension were retained. In each cluster flagged as problematic, 1 item—the independent item—was kept and all others—the dependent items—were discarded. The remaining items, apparently rid of any dependence, were fitted in a Rasch model again and the correlations among the residual values were checked anew to see if new clusters were formed, discarding items again, looping this way until no more problematic clusters were found.

Finally, with the LS/CMI purged of redundant items, its internal consistency was compared to its very own original internal consistency calculated with all 43 items. Similar comparisons were made for the predictive validity of the LS/CMI, calculated with and without redundant items. The test information curve of the LS/CMI was also produced with and without the items deemed redundant to verify if the removal of such items resulted in a considerable loss of psychometric information.

## Results

### Pearson Correlations

Four clusters of highly correlated items ( r≥.7 ) were found within the LS/CMI:

In cluster A, (Table 1), all items considered previous convictions. According to the LS/CMI instructions, item 1 is endorsed for an individual who has at least one past disposition/conviction, item 2 is endorsed if the individual has at least two past dispositions/convictions and item 3 is endorsed if the individual has three past dispositions/convictions or more. Thus, endorsing item 2 is, in fact, endorsing 2 items and endorsing item 3 is endorsing 3 items at once, a clear case of local dependence, especially when observing that the dependent items are endorsed 100% of the time.

**Table 1.** High Pearson Correlations Within the LS/CMI Items, Grouped in Clusters.

Items	Item 1	Item 2	Item 5	Item 9	Item 15	Item 16	Item 36	
Item 2	.74		Cluster A					
Item 3		.82						
Item 15				.89			Cluster C	
Item 16				.95	.92			
Item 17				.94	.92	.98		
Item 41			.76	Cluster B				
Item 42							.78	Cluster D

In cluster B, item 5 and item 41, although pertaining to different dimensions, were deeply related since, according to the LS/CMI instructions, endorsing item 41 is showing severe problems of adjustments in childhood or endorsing item 5, plus one of three conditions. Hence, when item 41 was endorsed, item 5 was endorsed 97% of the time.

Cluster C contained the most problematic correlations, its items seeming almost collinear with coefficients ranging between 0.89 and 0.98. However, finding high correlations among these items was not surprising knowing how they are scored. Following the LS/CMI instructions, when item 9 is endorsed (the individual being at school or working), items 15, 16, and 17 cannot be endorsed (0–0–0). Furthermore, when item 9 was not endorsed, items 15, 16, and 17 followed the same pattern (0–0–0 or 1–1–1) 88% of the time and items 16 and 17 (0–0 or 1–1) 97% of the time.

Cluster D comprised 2 items from different dimensions: item 36 and item 42. The Pearson correlation between the two was relatively high (.78) but the conditions determining when and how these items can be endorsed testified even more for a clear case of local dependence. According to the LS/CMI instructions, item 42 is automatically endorsed following the endorsement of one of items 36, 37 or 39 and it cannot be endorsed on its own. Also, when item 42 was endorsed, it was often because item 36 was endorsed in the first place, regardless of items 37 and 39’s endorsement.

### Rasch Modeling of the LS/CMI Data

LS/CMI data from Quebec’s male population of inmates was fitted in a unidimensional Rasch model, not with the intent to use or compare the difficulty parameters estimated for each item but with the intent to analyze how the Rasch residuals correlated passed the first dimension since highly correlated residuals do, indeed, point to local dependence (Lee, 2004; Yen, 1993). In Winsteps’ table 23.99 (Linacre, 2022b) residual correlations of 0.4 and higher were reported and used to identify items potentially dependent.

### Residual Correlations in Cluster A: Dispositions and Convictions

The correlations within the residuals of the first 3 items of the LS/CMI were, indeed, problematic, especially between items 2 and 3 (Table 2). Even with a more permissive threshold (e.g., .7), the correlation of the residuals of these items still pointed to local dependence. Since items 1, 2, and 3 were highly correlated at first and still seemed to be passed the Rasch dimension, a cut had to be made within this cluster^2^ to only keep the item whose endorsement prescribed others’.

**Table 2.** Residual Correlations in Cluster A.

Items	Item 1	Item 2
Item 2	.63	
Item 3	.46	.72

### Residual Correlations in Cluster B: Early Problematic Behavior

Not much changed between items 5 and 41 post Rasch modeling. Even with the main component dealt with, their correlation went from .76 (item correlation) to .65 (residual correlation). The dependence observed quantitatively and qualitatively at first was not weakened significantly with the Rasch dimension. Therefore, a cut had to be made within this cluster,^3^ only one item being sufficient to inform on a single declination of the latent trait (Table 3).

**Table 3.** Residual Correlation in Cluster B.

Items	Item 5
Item 41	.65

### Residual Correlations in Cluster C: Employment Issues

Cluster C was highly problematic at first and still seemed to be since the residuals were almost as correlated as the observed scores were, as if the Rasch dimension had no tangible effect on the forces that bind together specific items related to employment. For example, items 16 and 17 were correlated at .98 before Rasch modeling and their residuals were still correlated at .97 post Rasch modeling (Table 4). Consequently, one item out of the five had to speak for them all, others were discarded from the model,^4^ deemed too dependent or too redundant. Other residual correlations emerged post-Rasch modeling, with item 10—Frequently unemployed—correlated to items 9 and 16, but since item 10 didn’t seem to correlate too much at first, it was not considered as a candidate for removal.

**Table 4.** Residual Correlations in Cluster C.

Items	Item 9	Item 10	Item 15	Item 16
Item 10	.42			
Item 15	.82			
Item 16	.91	.4	.87	
Item 17	.90		.86	.97

### Residual Correlations in Cluster D: Criminal Attitude

Through Rasch modeling, cluster D saw another item surface, item 37—Unfavorable toward convention—flagged too strongly correlated to item 42 (Table 5). Thus, while accounting for some of the observed variance within the data, the Rasch dimension seemed to have stripped these items of what differentiated them. However, it was not surprising since, as mentioned before, item 42 is automatically endorsed following the endorsement of one of items 36, 37, or 39. Nevertheless, item 37 was not discarded since there was not enough evidence in the first place to justify its removal, whereas one of items 36 and 42 had to be dropped,^5^ the two of them deemed locally dependent with their residuals almost as highly correlated as the initial observations.

**Table 5.** Residual Correlations in Cluster D.

Items	Item 36	Item 37
Item 37		
Item 42	.67	.42

### Residual Correlations in Clusters E, F, and G

With the data fitted into a Rasch model, three additional clusters were formed among the residual correlations (Table 6). First, in cluster E, items 12—Less than regular grade 10 or equivalent—and 13—Less than regular grade 12 or equivalent—ended up highly correlated, although they should be. After all, item 12 could not be endorsed if item 13 was not endorsed first, even if some data showed such abnormalities.

**Table 6.** Other High Residual Correlations Within the LS/CMI Items.

Items	Item 12	Item 26	Item 28	Item 29	Item 30	Item 31	
Item 13	.45	Cluster E					
Item 27		.4	Cluster F				
Item 29							
Item 30			.58				Cluster G
Item 31				.46			
Item 32					.43	.47	

Next, in cluster F, the residuals of item 26—Few anticriminal acquaintances—and item 27—Few anticriminal friends—also correlated, but only slightly, right at the threshold (.4). The initial numbers didn’t flag these items as highly correlated (.52), but it was surprising, since these items were also clearly locally dependent, as item 27 must be endorsed for item 26 to be (but not vice-versa) according to the LS/CMI instructions.

Finally, in cluster G, 5 items from the sixth dimension of the LS/CMI—Alcohol/Drug problem—ended up highly correlated through their residuals after addressing the Rasch dimension. Naturally, items pertaining to drug abuse, namely item 29—Drug problem, ever—and item 31—Drug problem, currently—correlated well together and items pertaining to alcohol abuse, item 28—Alcohol problem, ever—and item 30—Alcohol problem, currently—correlated just as well. A quick analysis of the data showed that endorsing an item tied to a “current” problem with a substance prescribed the automatic endorsement of the corresponding “ever” item 100% of the time, another clear case of local dependence. Also, both “current” problems showed residual correlations with item 32—Law violations—one of the four options that must be endorsed when any “current” problem item is endorsed (items 32–35). Since an option must be chosen when item 30 or 31 is endorsed, there was dependence but not directly toward item 32, since other options could be selected. If item 32’s residuals ended up highly correlated with items 30 and 31’s, it was possibly because the option “Law violations” was chosen 87% of the time, regardless of other options. Despite the possible local dependence observed amidst the residuals within those three clusters, not enough evidence was gathered to warrant the purge of any item.

### Purging the Problematic Clusters of Their Local Dependence

All four clusters of highly correlated items identified before Rasch modeling ended up still correlated after Rasch modeling (Table 7). Within each cluster, 1 item had to be kept, ideally the one that best summed up the information they collectively provided. Several criteria were considered to determine which items to keep: the items that generated the most variance, the items that yielded the highest alpha coefficient, the items that seemed less correlated than others within each cluster. In the end, we chose to keep the independent items and not the ones that must be systematically endorsed following another’s. The item purge went as follows:

**Table 7.** Items Kept and Purged After the Analysis.

Clusters	Items kept	Items purged
Cluster A—Dispositions and convictions	Item 3	Item 1
		Item 2
Cluster B—Early problematic behavior	Item 41	Item 5
Cluster C—Employment issues	Item 9	Item 15
		Item 16
		Item 17
Cluster D—Criminal attitude	Item 36	Item 42

### Reliability, Predictive Validity, and Test Information Comparisons

Cronbach’s alpha was computed with all 43 items using JASP (JASP Team, 2022) and netted an impressive .902, a value well above the .7 standard. Such a value could be interpreted as excellent, but according to Laurencelle (1998, p. 152) a very high alpha index (e.g., higher than .9) could result from the presence of high correlations in an inter-item correlation matrix. Furthermore, this alpha value did not consider the fact that several items were known to be locally dependent, whose endorsement followed another’s (i.e., item 1 needing to be endorsed for item 2 to be endorsed, in turn needing to be endorsed for item 3 to be, and items 41, 42, 43, only endorsable if other items in different dimensions were endorsed), as specified in the user’s manual. Also, it did not take into account the fact that some items could only be endorsed in certain conditions (i.e. items 16 and 17, only endorsable for individuals that were employed or in school), some items measured very similar traits (items 24, 25, 26, and 27, pertaining to companions), or that 1 item was a constant (i.e. item 24, automatically endorsed for individuals that served a custodial sentence) yielding no useful information but still accounting for a portion of this “excellent” internal consistency. After withdrawing the seven locally dependent items, Cronbach’s alpha was calculated once more with the remaining 36 items and it still netted, 877, a value deemed very high but not “too” high.

Then, the predictive validity of the LS/CMI, when considering all its 43 items, was calculated using AUC, linking the individuals’ total scores to the criminal recidivism criteria, dichotomized as follows: 0 for non-recidivism and 1 for recidivism. With Quebec’s sample of detained men, the predictive validity of the LS/CMI reached .76, a value deemed high among actuarial tools. When calculating the predictive validity of the LS/CMI using the 36 locally independent items and not the seven redundant items that were purged, it reached .76 again. Thus, the LS/CMI seemed just as good at predicting criminal recidivism when using less items, but items that carry useful information, and not redundant information.

**Figure 1.**
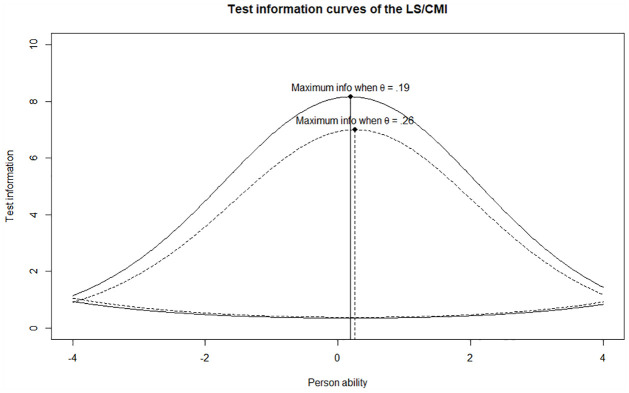
Test information and SEM curves of the LS/CMI.

Finally, test information was plotted with the original items of the LS/CMI (plain line) and the 36 items deemed independent (dashed line). Their corresponding standard error of measurement were also plotted, showing degrees of precision at different ability levels:

As expected, using more items—43 instead of 36—yielded more information, as the two plain curves show, but not necessarily enough to change the standard error of measurement significantly, as the two dotted curves show. Thus, using less items but “good” items did not decrease the precision of the LS/CMI significantly. Also, Figure 1 shows that the LS/CMI was most precise for individuals located near θ=.19 when all 43 items were used and near θ=.26 when the redundant items were not considered, a very small difference.

## Discussion

This paper examined a lesser-known psychometric evidence of the reliability of an actuarial tool by examining the effect of residual correlation structure analyses to better understand the internal structure of the LS/CMI.

### The Use of Alpha

First and foremost, it is important to note that there is less scientific literature on reliability than there is on the validity of actuarial tools. The most comprehensive references include Andrews et al. (2010) who compiled ten studies of the LS/CMI from different correctional jurisdictions and reported alpha coefficients ranging from .88 to .92 with an average of .89, a value consistent with that found in the present study.

Despite these studies, does not always constitute relevant psychometric evidence for assessing reliability. Bourque et al. (2020) argue that in most cases, other coefficients or estimators provide better insight than alpha. Cho (2016) is more critical, stating that the popularity and widespread use of alpha is not due to its metric superiority, but rather to better “marketing”: its name resonated well, its calculation was simpler, and it was a “coefficient” rather than an estimate, which gave the impression of being more precise or defined. Today, the use of alpha is criticized; it is often misused or misunderstood in the social sciences (Bourque et al., 2020; Cho, 2016; Cortina, 1993). This confusion even leads some to suggest its outright abandonment (Peters, 2014).

### Alpha’s Assumptions

To allow alpha to correctly summarize the reliability of a test like the LS/CMI, certain assumptions must be met (Béland et al., 2018). First, it must be assumed that the variance is identical for all test items. Yet, redundancy among items, as seen in the LS/CMI, reduces the variance of those items, yielding uneven variance across all items. A violation of this assumption may lead to an underestimation (Miller, 1995) of the actual reliability.

Furthermore, all items must be tau-equivalent, meaning they must have equal covariance, equal discrimination, and load equally on a 1-factor model of factor analysis. This assumption is very strong and hard to meet empirically. For example, Gordon et al. (2015) had to discard items 4, 11, 14, 21, 22, 29, and 40 in their analysis of the LS/CMI because they showed poor discrimination compared to others. Giguère et al. (2015) calculated the discrimination indices of the LS/CMI items with Quebec’s population of male inmates and probationers and reported poor discrimination, specifically for items in the Family/Marital dimension. Clearly, items do not discriminate similarly since some items seem very well correlated with the total score (e.g., criminal history) and others negligibly or not significantly.

A third assumption is that the data is unidimensional. However, the LS/CMI has eight theoretical dimensions and although the factor analysis of Giguère et al. (in press) of LS/CMI data from Quebec offenders and the factor analysis of Gordon et al. (2015) and Schmidt et al. (2016), did not yield the same latent structure as presented in Andrews et al. (2004), everything indicates that the LS/CMI is not based on a unidimensional model. Thus, Alpha’s second assumption regarding tau equivalence and the need to have equal factor loadings for all items is very difficult to satisfy. Additionally, while the data was deemed “sufficiently unidimensional” for certain analyzes to be performed, the tool is known to be multidimensional and is presented as such and reporting the alpha coefficients of a multidimensional instrument can be hazardous (Béland et al., 2018; Nunnally & Bernstein, 1994).

The use of alpha to summarize the reliability of the LS/CMI, or any other instrument, is questionable. In the same sense, judgments made on instruments according to their reliability, as reported by alpha, are not based on solid grounds if the alpha’s assumptions are not met. Nonetheless, if it was to be assumed that the LS/CMI was fit for the alpha to display its full efficiency, that the assumptions held, there would still be locally dependent items in the instrument. As shown in this study, these items would not provide anything unique or meaningful and they would not contribute to the “excellent reliability” of the LS/CMI.

### Possible Solutions

The present study has shown that items 1, 2, 5, 15, 16, 17, and 42 are redundant. Their endorsement often ends up being the result of an algorithm. Thus, the very structure of the LS/CMI constrains the officer completing it: they do not always have the freedom to endorse or not to endorse certain items. In other words, the endorsement of an item often leads to the endorsement of another. The justification for this redundancy is not in the LS/CMI user’s manual. Presumably, doing so gives more weight to specific elements. However, cloning information to give it more weight is difficult to justify in terms of psychometrics, especially when there are models that can handle such cases, and item response theory has many to offer. For example, the partial credit model (Masters, 1982), an extension of the Rasch model (Rasch, 1960), deals with polytomous responses as found in Likert scales (0–4), and a person’s ability can be estimated from responses to polytomous and dichotomous items in the same instrument. When the only possible responses are Success (1) or Failure (0), the PCM reduces to a Rasch model. The 2PL model (Birnbaum, 1968) considers a difficulty parameter and a discrimination parameter for each item. When estimating a person’s ability in this model, greater weight is given to items with good discrimination power. Finally, the graded response model (Samejima, 1969), like the PCM, deals with different possible responses, each with a specific associated score, while also considering a discrimination parameter for each item that gives them more or less weight in the estimation of a person’s ability according to their responses. When the only possible answers are Success (1) or Failure (0), the GRM reduces to a 2PL model. Thus, with so many models to choose from, the weighting could be done adequately, if that was indeed the purpose of this redundancy.

Another way around would be to follow the insight of Nunnally and Bernstein (1994) and report alpha for each construct/dimension separately. However, this would prescribe conducting a proper factor analysis on the items of the LS/CMI since the dimensions depicted in the manual are not based on statistical analyzes, as shown in the factor analysis of Gordon et al. (2015). In doing so, one of alpha’s assumptions would be more attainable, if not met.

Using another coefficient as an estimate of reliability could also be considered. Many turn to McDonald’s (1985, 1999) omega but there is also Guttman’s (1945) lambda-2, lambda-4, and lambda-6 and Woodhouse and Jackson’s (1977) GLB. Bourque et al. (2020) compared them all and throughout the multiple scenarios they experimented with, Omega and Lambda-6 were always superior to alpha, thus they recommended those two to estimate reliability, when available. However, using any other coefficient would still come with a set of assumptions to be checked. For example, “proper interpretation of omega [. . .] requires that the target model fits the empirical data well [and] parameter estimates need to be precise” (Brunner et al., 2012). Could we make sense of omega, knowing that the LS/CMI data must fit a one-factor model?

Finally, the 7 items deemed redundant could be removed from the instrument outright as they were in this study. The LS/CMI would be just as viable and reliable without these items while taking less time to complete, although alpha’s assumptions would still need to be met with the remaining items. Naturally, removing items from the LS/CMI would mean recalculating the thresholds used to classify individuals in the different risk categories. However, since those items are redundant and follow the endorsing patterns of other items within the scale, not much calculation would be required. Besides, the LS/CMI user manual provides little information on the process used to establish the risk levels. This classification is based on a simple aggregation of scores, which can lead to a loss of information. A study is underway with an innovative approach (Arbour et al., 2022) using random probability forests on a large number of LS/CMI assessments to calculate individual probabilities of reoffending while exploiting all the information contained in the risk assessments. This method produces better rankings, is simple to implement and can be used on different populations of prisoners and probationers.

One way or the other, dependence should be dealt with, as it was shown that having more items measuring the same subconstructs does not improve the internal consistency of the LS/CMI; the instrument clearly has no use for these items. Moreover, alpha’s assumptions or any other reliability coefficient’s assumptions should be checked before use, otherwise the values reported cannot be rightfully interpreted (Béland et al., 2018). In the present study, alpha was calculated without checking these assumptions, because we were not as interested in its value as in the change in its value when local dependence was handled.

The current study has a few limitations. First, it does not include individuals with heavy sentences and individuals serving a sentence in the community. Moreover, we do not know whether these results apply to women. Finally, this psychometric study was limited to general recidivism. The findings cannot be applied to violent or sexual offenses.

## Conclusion

Tools developed to assess offenders and identify both criminogenic needs and levels of risk for criminal recidivism must accumulate psychometric evidence pertaining to validity but also reliability. When the reliability of an instrument such as the LS/CMI is estimated through the internal consistency of its items, caution is advised since one cannot consider any coefficient as evidence of reliability if the assumptions underlying its use—variance, tau-equivalence and unidimensionality for alpha for example—are not respected.

Nevertheless, this study falsely assumed alpha could be considered evidence for reliability, fully aware that trying to interpret it would be hazardous. However, the objective was not to give meaning to alpha but to study the effects of local dependence within the items of the LS/CMI on the alpha coefficient. Hence, CTT and IRT were used to identify redundant items: items whose correlations with other items were considered high before and after Rasch modeling were flagged “locally dependent.” Then, internal consistency, predictive validity and information were estimated with all 43 items and without the six dependent items. The results of the analyzes of this study showed the little effect that these items had on internal consistency. The analyzes also showed that these items did not strengthen the predictive validity of the LS/CMI. Furthermore, the psychometric information did not appear to provide any significant gain in the accuracy of the actuarial tool. In sum, although they may seem to contribute by their sheer number to a high alpha coefficient, the redundant items do not contribute anything useful from a psychometric point of view. The LS/CMI includes many of them so it could benefit from our results. If it was to be shortened for administrative or practical reasons, the items we identified as redundant should be further analyzed and reviewed by criminal risk management experts since these items clutter up the assessment process without providing any utility on case management and risk scoring.

We understand that the LS/CMI was constructed with the intention of introducing locally dependent items to give greater weight to certain risk factors—for example, 3 items related to prior convictions in the criminal history dimension, or 2 items related to friends and acquaintances in the companions dimension. In other fields, as, for example, in personality tests, it is common practice to have many items with different wordings but addressing the same trait to obtain a reliable measure of the said trait. This redundancy not only lends weight to certain traits, but first and foremost, it validates an individual’s response pattern. However, this redundancy does not seem useful within the context of the LS/CMI, the independent items seem reliable and show great validity. On the other hand, what we find more questionable from a psychometric standpoint, is the systematic endorsement of an item following another item’s endorsement, while both items do not seem to pertain to the same risk factor, resulting in the generation of error (Boudjemad & Gana, 2009). The LS/CMI contains many items whose endorsement is calculated according to different items pertaining to different dimensions. Those items are not endorsed according to an individual’s inherent trait, they are endorsed according to other items. Thus, they are not well aligned with the theoretical concepts they are meant to represent.

In summary, if local dependence does not contribute significantly to the reliability of a scale, does not contribute the predictive validity of a scale, and lacks solid theoretical justification, why have locally dependent items at all? It is in the interests of correctional systems to use efficient tools that can assess offenders within the allotted time while establishing a precise level of risk to facilitate the management of offenders and public safety. This pursuit of an efficient assessment process does not hinge on local dependence.

## Notes

1. The curious reader is referred to Giguère et al. (2022).

2. See the section “Purging the problematic clusters of their local dependence.”

3. See the section “Purging the problematic clusters of their local dependence.”

4. See the section “Purging the problematic clusters of their local dependence.”

5. See the section “Purging the problematic clusters of their local dependence.”
